# Impact of Remote Symptom Monitoring With Electronic Patient-Reported Outcomes on Hospitalization, Survival, and Cost in Community Oncology Practice: The Texas Two-Step Study

**DOI:** 10.1200/CCI.23.00182

**Published:** 2023-10-28

**Authors:** Debra A. Patt, Amila Meera Patel, Arun Bhardwaj, Kathryn Elizabeth Hudson, Amanda Christman, Ninad Amondikar, Susan Marie Escudier, Sydney Townsend, Holly Books, Ethan Basch

**Affiliations:** ^1^ Texas Oncology, Dallas, TX; ^2^ Navigating Cancer, Seattle, WA; ^3^ UNC Lineberger Comprehensive Cancer Center, Chapel Hill, NC

## Abstract

**PURPOSE:**

There is raising interest to implement electronic patient-reported outcomes (ePROs) for symptom monitoring to enhance the quality of cancer care. Step 1 of the Texas Two-Step Study demonstrated successful implementation of an ePRO system in >200 sites of service of a large community oncology practice. We now report step 2 of this study which evaluates the impact of ePROs on outcomes among patients enrolled in the Centers for Medicare & Medicaid Services' Oncology Care Model (OCM) program.

**METHODS:**

This observational study focused on patients with metastatic cancer enrolled in OCM at large community oncology practice located in Texas between July 2020 and December 2020. Patients who completed ≥1 survey via the ePRO tool were included in the study group and were propensity score matched with patients in a control group. Adverse events (AEs; hospitalizations, emergency department visits, deaths) and total cost of care were a priori study outcomes. Mann-Whitney *U* and chi-square tests compared continuous and categorical variables, respectively, with multivariable logistic regression for adjustment of covariates.

**RESULTS:**

Of 831 patients with metastatic cancer, 458 matched patients (229/group) were identified, with 52% male and a mean age of 74 years. Mean total AEs were lower in the study group compared with control (0.98 *v* 1.41; *P* = .007), with decreased hospitalizations (20% *v* 32.5%; *P* = .002), emergency visits (38.4% *v* 42.3%; *P* > .05), and deaths (11.8% *v* 16.6%; *P* > .05). Average number of hospitalizations was lower (0.28 *v* 0.52; *P* = .003) with reduced mean duration of hospitalizations (1.9 vs 3.2 d; *P* = .03). The total cost of care was reduced by an average of $1,146 per member per month.

**CONCLUSION:**

Symptom monitoring with ePROs improved quality and value of cancer care delivery by reducing hospitalizations, emergency visits, and deaths while lowering cost of care in a large oncology practice.

## Teaser

The Texas Two-Step Study demonstrates #ePROs improve outcomes and reduce costs for patients with advanced cancer.

## BACKGROUND

Patients with cancer can experience debilitating symptoms related to disease and treatment which can contribute to a diminished quality of life, emergency room visits, hospitalizations, and even early death.^1,2^ Electronic patient-reported outcomes (ePROs) provide a real-time option for symptom monitoring that can facilitate rapid intervention to optimize symptom control.

**CONTEXT**

**Key Objective**
To characterize the benefit in patient outcomes and total cost of care of electronic patient-reported outcome (ePRO) implementation across a large community oncology practice.
**Knowledge Generated**
Implementation of ePROs did reduce adverse events (AEs; emergency room visits, hospitalizations, and death) among Medicare beneficiaries compared with controls and reduced the total cost of care by almost 10%.
**Relevance *(J.W. London)***
By implementing ePRO for symptom monitoring this study demonstrates that the incidence of patient AEs is lower, reducing patients' cost of care.*
  *Relevance section written by *JCO CCI* Associate Editor Jack W. London, PhD.

Symptom management optimization improves patients' quality of life, adherence to medication, and overall survival as demonstrated in previous studies in academic centers, population research, and in limited enrollment studies in the community.^3^ There is increasing interest in broad implementation of ePROs in community oncology practice but limited published demonstrations of feasibility or impact on outcomes in this setting. Therefore, we sought to implement ePROs in a large multisite community oncology practice and evaluate the outcomes of that intervention to understand if these tools could have a similar effect in a multisite community oncology setting. Implementation was successful as previously published, and this evaluation characterizes outcomes.^4^ This study seeks to measure the impact of ePROs on emergency room (ER) visits, hospitalizations, death, and total cost of care. This evaluation is in the context of multiple measures implemented across the practice during the period of the Center for Medicare Medicaid and Innovation Oncology Care Model (OCM) and within the first year of the COVID-19 pandemic.

## METHODS

### Study Design and Data Sources

**FIG 1.**
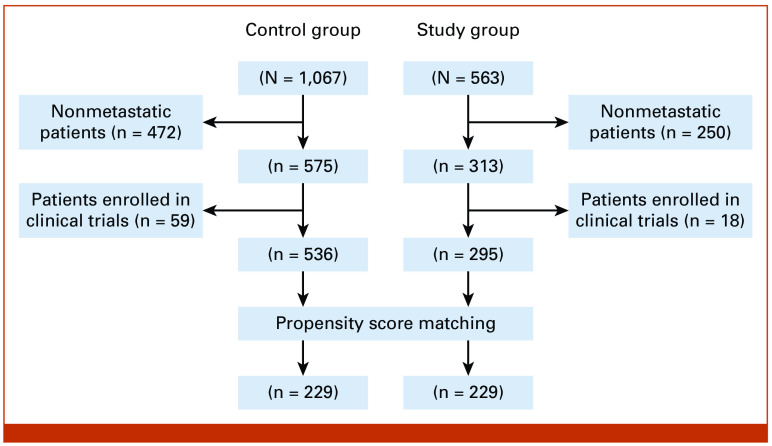
Flow diagram showing the selection of control and study patient cohorts.

The objective of this observational study was to compare the adverse events (AEs) and cost of care among patients who participated in an ePRO remote monitoring program to track symptoms and oral medication adherence against a matched patient cohort who did not (Fig 1). Included patients were undergoing systemic treatment for metastatic cancer and were enrolled in the OCM program from the Centers for Medicare & Medicaid Services (CMS) between July 2020 and December 2020 at multiple sites of service across large community oncology practice located in Texas. Large community oncology practice located in Texas is a large US statewide community oncology practice, which has implemented multiple digital health solution care enhancements, including an ePRO tool using HIPAA-compliant software and implementation support from Navigating Cancer (NC). Patients enrolled in OCM were selected for inclusion in this analysis because they have Medicare claims data available as a part of the OCM program that were available for the planned analyses as outcomes. A research data set was constructed for this analysis consisting of OCM claims data derived from large community oncology practice located in Texas, linked to data collected by NC's ePRO software system. The large community oncology practice located in Texas claims data for this analysis included demographic variables and clinical data elements. Owing to small sample sizes in individual cancer type categories, cancer type apart from breast, lung, chronic leukemia, lymphoma, small intestine/colorectal, multiple myeloma was grouped as other. This study was reviewed by the internal privacy review board and exempted from institutional review board approval as it was a part of a quality improvement process throughout the entire practice. Patients participated in informed consent when platform use initiated.

### Propensity Score Matching

To balance the confounders between control and study groups, propensity scores using logistic regression were estimated, and matching was performed using a nearest-neighbor matching algorithm with replacement^5^ to minimize selection bias after adjusting for age, sex, cancer type, radiation, surgery, and line of therapy. Standardized mean differences (SMDs) were calculated and compared before and after matching for all variables to assess the covariate balancing. A SMD <0.02 was considered as an indicator of successful balancing.^6^ After finding the matched population, propensity score overlapping was performed to verify the common support,^7^ and *t*-test and chi-square testing were conducted to evaluate differences in continuous and categorical variables, respectively, between matched groups.

### Study Measures

Prespecified outcomes for this analysis included total AEs (defined below), hospitalization rate, ER visit rate, and death rate during a single 6-month episode of care for OCM (ie, July 2020 to December 2020). Total AEs for each patient were calculated by adding the total number of hospitalizations and total number of ER visits to the patient's deceased status (0 for alive and 1 if deceased) during 6 months of the study period. Rate of total AEs, hospitalization, and ER visit was calculated as 100 per person days. In addition, total Medicare cost per member per month (PMPM) was calculated using Medicare actual paid amounts for care delivery services (ie, outpatient, inpatient, home health agency [HHA], hospice [HSP], physician (PHY), durable medical equipment [DME], skilled nursing facility [SNF]) and compared between the study arms. In the case of inpatient services, the total amount paid by Medicare was based on the sum of inpatient claim payment amounts and inpatient Medicare-covered utilization amounts (calculated by multiplying the daily per diem amount by the number of Medicare-covered utilization days).

### Statistical Analysis

Continuous variables were expressed as mean ± standard deviation unless otherwise indicated and were compared using the two-sided student *t*-test. Chi-square testing was used to compare categorical variables. Multivariable logistic regression models were constructed and used to adjust for baseline demographic and clinical characteristics in analyses of rates of hospitalization, ER visits, and deaths in the studied cohort. Characteristic factors in each comparison were expressed as odds ratios with 95% CIs. Statistical significance was defined as a *P* value <.05. Analyses were performed in Python (version 3.8.5), with R-package ggplot2 used to visualize output of logistic models.

## RESULTS

### Patient Demographics

Of initial 1,630 patients with cancer identified, approximately 51% (n = 831) met the selection criteria for this study (Fig 1) which comprised 536 control and 295 study group patients. In the unmatched population, the average age of patients in the control and study cohort was similar at about 74 years. Before matching, the proportion of patients (control *v* study) was significantly different on cancer type, radiation, and therapy line categories. The study group was more likely to have lung or colorectal cancer, receiving first-line therapy and radiation treatment as compared with the control group (Table 1), which was in accordance with the significantly different average propensity score between groups (0.314 and 0.447 in control and study, respectively; data not shown). Of 831 patients, 458 matched patients with cancer (n = 229 patients per group) were identified through propensity score matching, with a propensity score–matched score which was similar (approximately 0.418) in each arm of the matched cohort, indicating successful balancing of confounders among experimental groups. Patient distribution of the matched control and study cohorts was similar among all focused variable categories with no significant difference using chi-square analysis. In our matched cohort, approximately 52% of patients were male, with a mean age of 74 years. The majority of matched patients in the control and study groups had other cancer types (>30%), did not receive radiation (approximately 89%) or surgery (approximately 97%), and were on first line of therapy (>55%; Table 1).

**TABLE 1.**
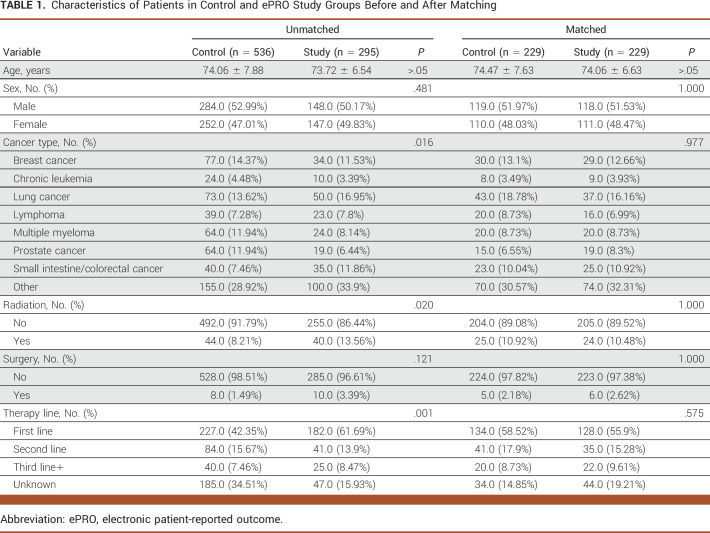
Characteristics of Patients in Control and ePRO Study Groups Before and After Matching

### ePRO Participation and Reduced Adverse Events

**FIG 2.**
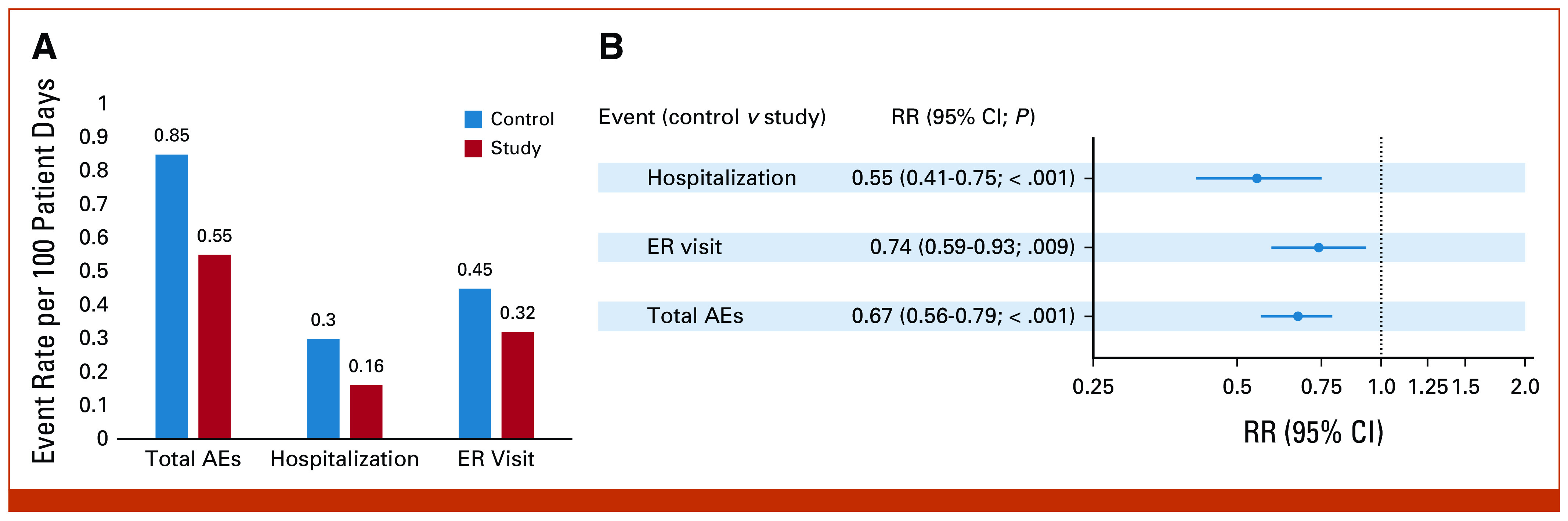
Associations of ePRO participation with AEs. (A) Bar graph showing the rate of total AEs, hospitalizations, and ER visits in the control and study groups. Data show the rate per 100 person days. (B) RR between control versus study groups was calculated using the Poisson regression model and plotted with 95% CIs in the forest plot. AEs, adverse events; ePRO, electronic patient-reported outcomes; ER, emergency room; RR, rate ratio.

To investigate the impact of the ePRO remote symptom monitoring program on patient outcomes, we first calculated the total AEs (sum of total hospitalizations, ER visits, and deaths) that occurred per 100 patient days during the study period and compared the matched control versus study groups. We observed approximately 33% fewer AEs (total AEs: 0.85 and 0.55 per 100 person days in the control and study group, respectively; Fig 2A) in patients who participated in the ePRO study group as compared with control (rate ratio [RR], 0.67 [95% CI, 0.56 to 0.75]; *P* value < .001; Fig 2B). To characterize underlying drivers of ePRO participation-mediated reduced AEs in patients with cancer, we compared total hospitalizations and ER visits between the control and study groups. We found fewer visits for patients with cancer in the ePRO study group compared with controls for hospitalization (hospitalization rate 0.3 and 0.16 per 100 per days in the control and study group, respectively; RR, 0.55 [95% CI, 0.41 to 0.75]; *P* < .001; Figs 2A and 2B) and ER visits (ER visit rate in control: 0.45 *v* study: 0.32, Fig 2A; RR, 0.74 [95% CI, 0.59 to 0.93]; *P* = .009; Fig 2B).

**FIG 3.**
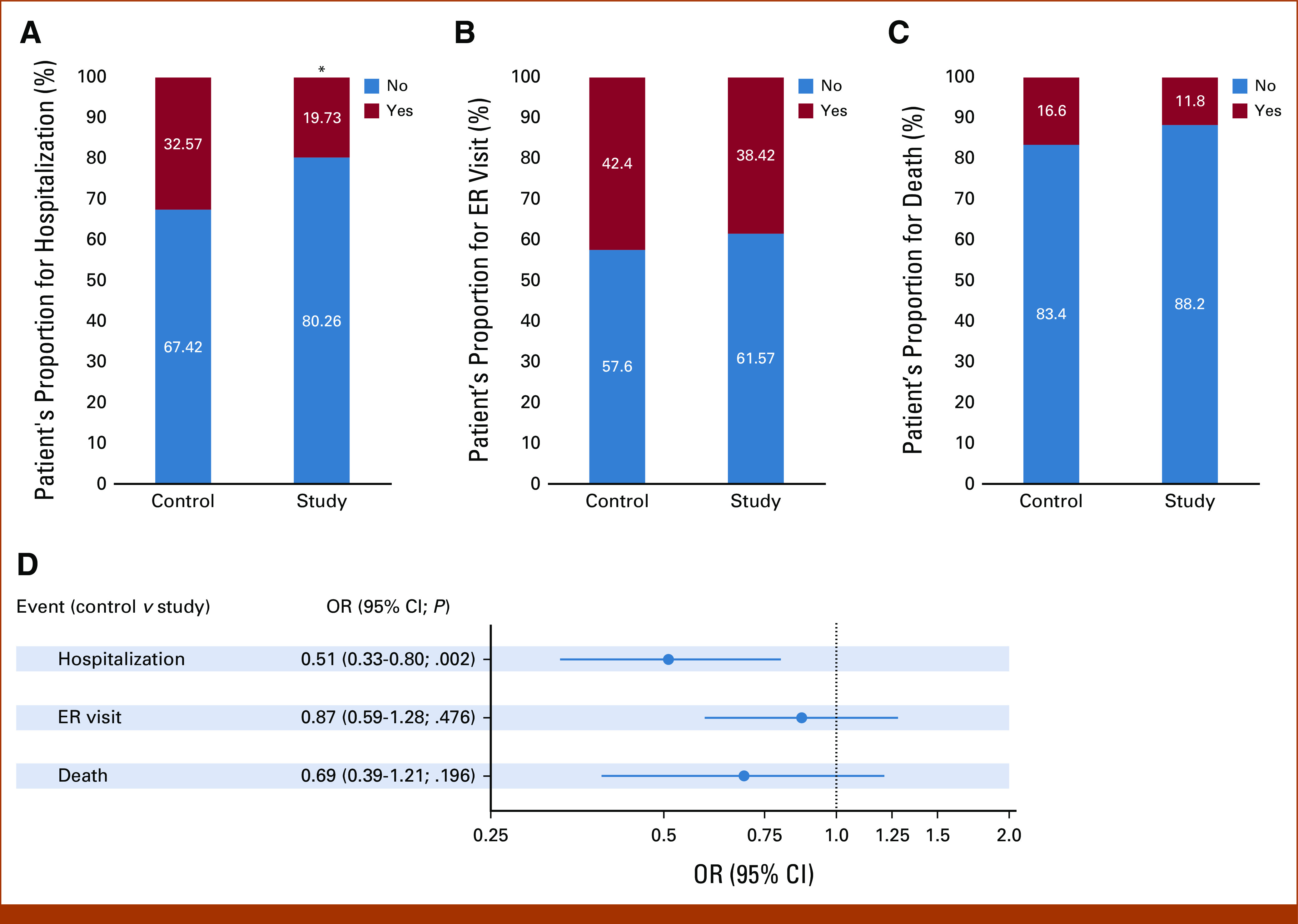
Proportion of hospitalization, ER visit, and deaths among study versus control groups. Figure depicts the proportion of patients who were (A) hospitalized, (B) visited ER, and (C) deceased in the control versus study group during the study period. A chi-square test was performed to compare the difference. *P* value < .05 considered statistically significant. (D) Logistic regression model was constructed to control the different covariates as mentioned in Methods, and adjusted odds were calculated for hospitalization, ER visit, and deaths in control versus study populations. Results are shown as aORs with 95% CIs. aOR, adjusted odd ratio; ER, emergency room; OR, odds ratio.

Additionally, the proportion of patients with each AE (hospitalization, ER visits, and death) in the control and study groups was calculated and compared using a chi-square test (Figs 3A-3C). There was a significant association between hospitalization and ePRO participation in patients with cancer, as the study group patients were hospitalized less than controls (32.6% and 19.7% in the control and study group, respectively, *P* < .05; Fig 3A). We observed decreased but not significant proportions of ER visits (38.4% *v* 42.3%; Fig 3B, *P* value > .05) and deaths (11.8% *v* 16.6%; Fig 3C, *P* value > .05) in the study group compared with control.

After adjusting for covariates, patients within the study group had significantly decreased odds of hospitalization during the study period as compared with the control group (adjusted odd ratio [aOR], 0.51 [95% CI, 0.33 to 0.8]; *P* value .002; Fig 3D). Similar, but not statistically significant, effects were observed for ER visit and death rates. The study group was less likely to be deceased (aOR, 0.69 [95% CI, 0.39 to 1.21]; *P* > .05) and visit the ER compared with the control group (aOR, 0.87 [95% CI, 0.59 to 1.28]; *P* > .05; Fig 3D).

### ePRO Program Associated With Lower Total Cost of Care

**FIG 4.**
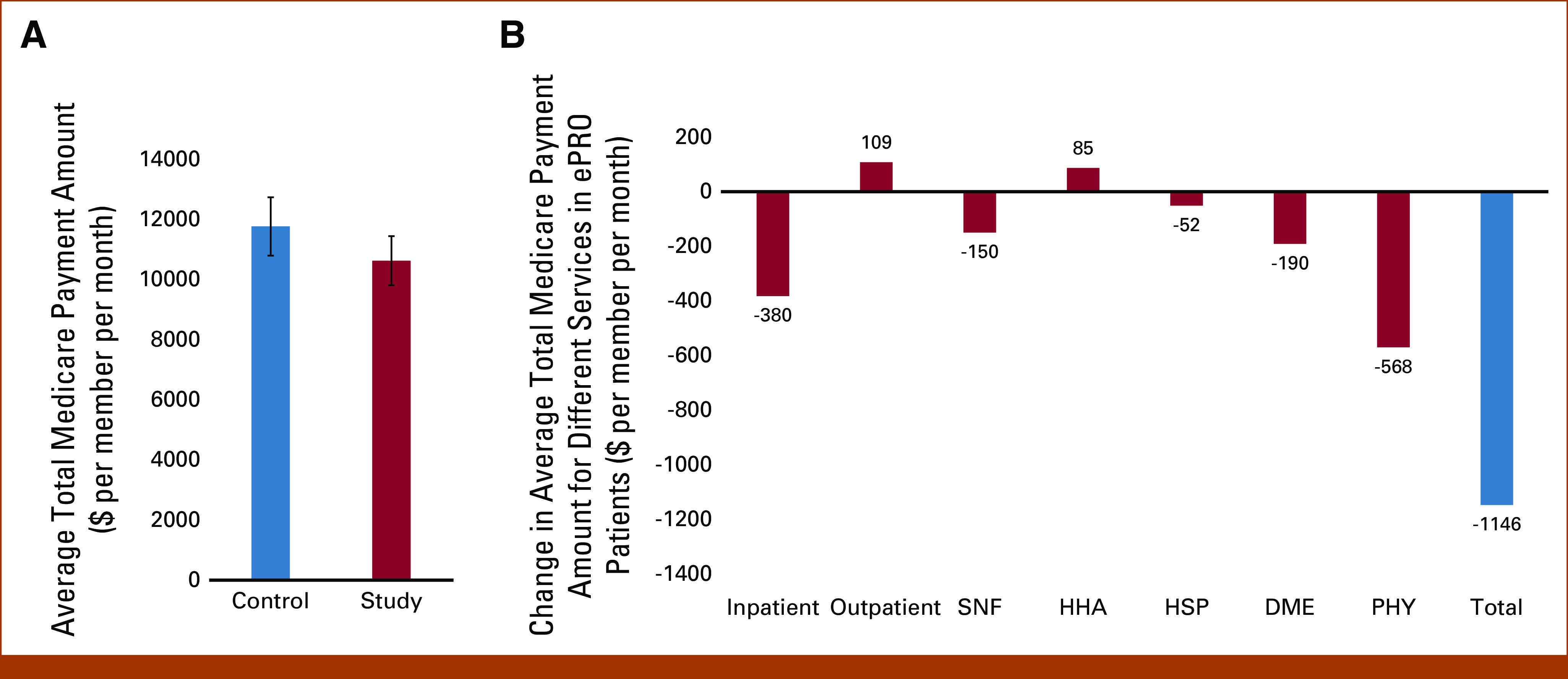
Association of ePRO engagement with total cost of care. (A) The total Medicare payment amount PMPM in the control versus study group. Data are shown as mean ± SE, and *P* value < .05 is considered statistically significant. (B) Bars represent the difference in average total Medicare payment amount PMPM for different services in the study group as compared with control. DME, durable medical equipment; HHA, home health agency; HSP, hospice; PHY, physician; PMPM, per member per month; SNF, skilled nursing facility.

As shown in Figure 4A, the total Medicare reimbursement for patients who participated in the ePRO program was $10,624 per month per member which was approximately 10% ($1,146) less than the control group ($11,770). Next, to understand the service type(s) that were major contributors to the lower total cost of care in the study group, we compared the Medicare payment amounts for each service. In comparison with the control group, we observed decreased total Medicare cost associated with inpatient, SNF, HSP, DME, and PHY services in the study group. However, an opposite pattern was seen for outpatient and HHA costs (Fig 4B).

The total Medicare paid amount for PHY services was identified as the largest contributor to decreased costs in the ePRO study group, with a difference of approximately $568 PMPM compared with controls (Fig 4B). The total Medicare payments for inpatient, DME, and SNF services were $380, $190 and $150 less in the study group compared with controls, respectively. Contrary to other services, total Medicare paid amounts were higher for outpatient and HHA services ($109 and $85 PMPM, respectively) for patients who participated in the ePRO program compared with controls (Fig 4B).

## DISCUSSION

In this study of ePRO implementation across a large multisite community oncology practice, significant benefits were observed in hospital utilization and cost of care. This finding adds to the mounting evidence of benefits of ePRO symptom monitoring in oncology and demonstrates that these benefits are conferred in the community oncology setting when widely implemented. These findings are particularly salient for value-based care models such as the CMS Enhancing Oncology Model (EOM), in which ePRO collection is a required care enhancement. Overall, the Texas 2-Step study shows the feasibility and benefits of wide implementation of ePROs in community oncology practice.

It is important to note that this study occurred amid a landscape that made multiple investments in value-based care over decades and implemented many operational processes guidelines and tools to improve care quality that could influence the control and intervention group. For more than 15 years, the practice has participated in evidence-based pathways to optimize patient outcomes while lowering costs.^8^ The practice implemented triage pathways and set a standard that patient symptoms are addressed rapidly. Hiring additional nurse navigators, social workers, financial counselors, and palliative care clinicians were investments in the cultivation of services to enhance patient care. Treatment care plans and digital education on the patient portal were developed to enhance communication with patients. Nurse triage systems to shorten response time to symptom control with management pathways were implemented. Within this value landscape, the practice saved the Medicare program substantial costs over nine performance periods, and hospital admissions declined by 9% and emergency room visits declined by 6% in the OCM program. The benefits we observe in this study address the incremental impact of ePRO use up and above the benefits realized within the entire OCM population, but infrastructure in place may have facilitated optimization of ePRO implementation. This demonstrates a profound impact of ePROs even within a fertile landscape of value-based health care delivery.

There are a number of limitations to this study. The observation of 33% fewer AEs should be evaluated more closely. The relative risk of hospitalizations was 0.55 (95% CI, 0.41-0.75) while the relative risk of ER visits was 0.74 (95% CI, 0.59-0.93) as one might expect that an intervention that managed symptoms earlier might decrease ER visits more than hospitalizations. This may have occurred for a number of reasons. Utilization of the emergency room and the hospital was affected by the COVID-19 pandemic, and this could have affected the intervention and control arms. Although total costs were $1,146 (about 10%) less in the intervention group versus the control group, higher costs were driven by higher costs for hospitalization and skilled nursing facilities for the control group while costs were higher for outpatient services in the intervention group. This may reflect efforts to manage early interventions within clinic or telehealth clinic visits in attempts to control symptoms early.

Some aspects of implementation also warrant further discussion. In our program, symptom management through ePROs is managed initially by nurses between office visits. Nursing leadership set an expectation of rapid symptom attention in real time. Leadership frequently evaluated and adjusted operation of the program to maximize rapid communication while taking care to manage nurse workload.

Given the large number of patients in the practice, the matched populations of patients with advanced cancer was relatively small. There also may be differences in the intervention and control groups, which could introduce bias. Understanding the impact of rural living, socioeconomic status, pain control, and palliative care would be useful but limited by a small study size and use of administrative claims. In addition, this study was conducted during the COVID-19 pandemic which could variably affect results. Despite study limitations, there is compelling evidence that these interventions enhance the value of health care delivery, and their broad implementation will improve patient outcomes.

The landscape of quality and value-based care will continue to evolve, and ePROs are a meaningful way to enhance the quality and value of patient care. As noted above, in 2023, CMS launched the Enhancing Oncology Model (EOM), a volunteer value-based payment model for patients with cancer within the Medicare program.^9^ This model requires certain quality enhancing tools such as ePROs as part of the platform. After the EOM pilot is completed, if successful, many of these enhancements in care delivery may become the standard of care in cancer care delivery.

In conclusion, implementation of symptom monitoring with ePRO was feasible and conferred significant benefits on hospital utilization and cost of care in a large multisite community oncology practice.
